# Far‐infrared sauna exposure at 65°C elevates core temperature

**DOI:** 10.1113/EP094028

**Published:** 2026-07-31

**Authors:** Elliott J. Jenkins, Joseph A. Killick, Sally R. Grimm, Sam R. Davies, Jemima A. Benson, Joshua C. Tremblay, Mike Stembridge

**Affiliations:** ^1^ Cardiff School of Sport and Health Sciences Cardiff Metropolitan University Cardiff UK

**Keywords:** core temperature, heat stress, sauna

## Abstract

Far‐infrared (FIR) saunas are increasingly used as a passive heating and ‘wellness’ modality, yet their capacity to elevate core temperature remains unclear, particularly given prior work at lower temperatures (∼45°C) reports limited net heat storage. We therefore examined whether FIR sauna exposure at an ambient temperature more typical of traditional sauna bathing (65°C) increases core (rectal) temperature and evokes thermoregulatory, cardiovascular, haematological and perceptual responses associated with heat strain. Twelve healthy adults (6 female) completed a single 45‐min FIR sauna exposure. Rectal temperature, skin temperature, heart rate, blood pressure, plasma volume changes and perceptual measures were assessed throughout. Rectal temperature increased by 1.4°C (95% CI: 0.9, 1.8; *P *< 0.001), with elevations evident from ∼20 min. Skin temperature rose rapidly (+7.4°C [95% CI: 5.4, 9.5] at 5 min; *P *< 0.001) and remained elevated thereafter. Heart rate increased from 74 ± 15 to 153 ± 20 bpm (*P *< 0.001), while mean arterial pressure decreased immediately post‐exposure (−9 mmHg [95% CI: −2, −16]; *P *= 0.012), driven by reductions in diastolic pressure. Plasma volume contracted by −11.6% (95% CI: −15.9, −7.2; *P *< 0.001), with a sweat rate of 1.4 ±  0.5 L h^−^^1^. Thermal sensation and discomfort increased to ‘extremely hot’ and ‘extremely uncomfortable’, respectively (*P *< 0.001). In summary, FIR sauna exposure at 65°C elicits substantial increases in rectal temperature and systemic physiological strain comparable to values previously reported in the passive heating literature. These findings indicate that the effectiveness of FIR sauna to increase core temperature is governed by the thermal load imposed, supporting its potential as a heat‐based intervention when applied at sufficiently high temperatures.

## Abstract (highlights)

- **What is the central question of this study?**
  Does 45 min of far‐infrared (FIR) sauna exposure at a higher ambient temperature (65°C) provide a sufficient thermal stimulus to increase core (rectal) temperature in healthy adults?
- **What is the main finding and its importance?**
  Forty‐five minutes of FIR sauna exposure at 65°C increased rectal temperature by ∼1.4°C and evoked thermoregulatory, cardiovascular, haematological and perceptual responses associated with substantial heat strain. These findings demonstrate that the effectiveness of FIR sauna to increase core temperature is governed by the thermal load imposed, supporting its potential utility as a heat‐based intervention.

## 1 INTRODUCTION

Passive heating strategies, including sauna bathing and hot‐water immersion, are widely used for relaxation, recovery and to induce physiological adaptations relevant to cardiovascular and metabolic health (Laukkanen & Kunutsor, 2024; Laukkanen et al., 2015). These responses are largely mediated by elevations in core body temperature, which perturb thermal homeostasis and necessitate thermoregulatory and cardiovascular adjustments, including increases in skin blood flow, cardiac output and sweat rate (Crandall & Gonzalez‐Alonso, 2010; Periard et al., 2021). As such, changes in core temperature are considered a key determinant of the physiological and perceptual strain imposed by passive heat exposure (Cabanac & Massonnet, 1977), and are essential for driving the adaptive responses associated with heat acclimation (McDonald et al., 2025).

Traditional (Finnish) sauna typically exposes individuals to high ambient air temperatures (∼80–100°C, 15–20% relative humidity; Heinonen & Laukkanen, 2018), resulting in consistent and substantial (∼1–2°C) increases in core temperature (Leppäluoto et al., 1986; Zalewski et al., 2014). In contrast, far‐infrared (FIR) saunas deliver heat via radiant energy emitted from infrared panels, typically within a wavelength range of ∼6–14 µm (Qin et al., 2024), and are generally operated at lower ambient temperatures (∼45–60°C; Beever 2009; Reed et al. 2025). While traditional saunas rely largely on convective heat exchange, FIR systems additionally deliver radiant energy that is primarily absorbed by water‐containing tissues, contributing to localised heating of the skin and superficial tissues (Richey et al., 2026). However, in practical settings, particularly at higher operating temperatures, heat transfer within FIR saunas is unlikely to be exclusively radiative, as elevated ambient temperatures may also promote convective heat gain and influence evaporative heat loss. Despite their increasing popularity as a perceived wellness modality, and given their typically lower operating temperatures, it remains unclear whether FIR sauna exposure provides a sufficient thermal stimulus to meaningfully elevate core temperature and induce comparable physiological strain.

Recent work has reported marked increases in skin and skeletal muscle temperature during FIR sauna exposure, with muscle temperature increasing across depths of ∼1–3 cm beneath the skin surface in a depth‐dependent manner (i.e., greater increases in superficial vs. deeper tissues) (Reed et al., 2025). These responses occurred in the absence of an increase in core temperature (Δ: 0.0°C; intestinal), suggesting that FIR exposure predominantly heats peripheral tissues without substantially elevating central thermal load. Atencio et al. (2025) similarly reported modest physiological responses during a 45‐min FIR sauna exposure (e.g., ∼0.4 kg sweat loss, ∼1.6 L min^−^^1^ increase in cardiac output), again without appreciable changes in core temperature (Δ: 0.0°C; rectal or intestinal). This dissociation raises uncertainty regarding the efficacy of FIR saunas as a passive‐heating modality, particularly given the established role of core temperature in shaping physiological responses to heat exposure. Importantly, these findings are largely derived from protocols conducted at ∼45°C, where the overall thermal load – and thus the combined contributions of radiative, convective and evaporative heat exchange – may have been insufficient to meaningfully elevate whole‐body heat storage. As such, the extent to which higher‐temperature FIR sauna exposure elicits meaningful increases in core temperature, and associated cardiovascular and perceptual strain, remains unclear.

Given that the physiological responses to passive heating depend on the intensity and duration of the thermal load, it is plausible that FIR sauna exposure at higher ambient temperatures may provide a more substantial stimulus than previously reported. However, this has not been systematically examined. Therefore, we examined whether FIR sauna exposure at 65°C increases core (rectal) temperature in healthy adults, alongside associated thermoregulatory, cardiovascular and perceptual responses. We hypothesised that 45 min of FIR sauna exposure at 65°C would increase rectal temperature by ≥0.5°C and elicit concomitant increases in thermoregulatory, cardiovascular and perceptual strain.

## 2 METHODS

### 2.1 Ethical approval

Study procedures were approved by the Cardiff School of Sport and Health Sciences Ethics Committee (Sta‐12386) and conformed to the ethical standards established by the *Declaration of Helsinki* (2013), excluding prior registration in a database. Written informed consent was obtained from each participant before testing.

### 2.2 Study design

Participants attended a single laboratory visit. Upon arrival, participants were instrumented (described below) and seated for a 15‐min baseline period in an ambient temperature room (∼21°C), after which baseline measurements were recorded. Participants then entered a FIR sauna (∼65°C) and remained seated for 45 min, with end‐of‐heating measurements obtained <3 min upon completion. Following sauna exposure, participants sat back in the ambient environment for 30 min, after which recovery measurements were recorded and participants were de‐instrumented.

### 2.3 Participants

Twelve healthy adults were recruited to participate in the study (6 male, 6 female; age = 26 ± 4 years; height = 176 ± 9 cm; body mass = 67.9 ± 9.9 kg). A priori estimation (α = 0.05, power = 0.80) indicated that a minimum of six participants were required to detect a 0.5°C increase in rectal temperature (*T*_REC_); recruitment was increased to 12 to ensure complete datasets. All participants were non‐smokers and free from known cardiovascular or metabolic disease. Participants were recreationally active and met standard safety criteria for heat exposure.

### 2.4 FIR sauna

Participants were seated in a FIR sauna (Sanctuary 2 Person Sauna Pro; Clearlight, Berkeley, CA, USA) for 45 min at a target temperature of 65°C. The sauna was switched on 1 h prior to each trial, and participants entered once ambient temperature exceeded 60°C (minimum entry threshold) to ensure a consistent thermal stimulus. The unit (interior dimensions: 118 × 112 × 186 cm; bench space: 114 × 56 cm) was equipped with True Wave® low EMF/ELF full‐spectrum heaters (2 × 700 W) located on the front wall, in addition to carbon/ceramic FIR heaters positioned on the back wall, side walls, under the bench and in the floor. Participants wore minimal clothing (bathing suits) and did not consume fluids during the exposure. Ambient temperature (TM20; Extech Instruments, Hudson, NH, USA) and relative humidity (Hygrometer 608‐H2; Testo, Titisee‐Neustadt, Germany) were measured using probes positioned at head height within the sauna.

### 2.5 Experimental procedure and measurements

Before each trial, participants were instructed to arrive euhydrated and to refrain from strenuous exercise for at least 6 h. Upon arrival, participants provided written informed consent and had their height measured. Participants then moved to a private area where a urine sample was obtained to assess hydration status (urine specific gravity < 1.020; Master‐URC/NM; Atago, Tokyo, Japan), nude body mass was recorded (RD‐545HR; Tanita, Tokyo, Japan), and a rectal thermistor (YSI 400 series, 9 Fr; PROACT Medical, Corby, UK) was self‐inserted to a depth of 15 cm for continuous *T*_REC_ monitoring (Squirrel SQ2010; Grant Instruments, Cambridge, UK).

Participants were then instrumented for the measurement of heart rate (HR; HRM‐Pro; Garmin Ltd, Olathe, KS, USA) and skin temperature (*T*_SK_; 2.3K3A1B NTC thermistors; Betatherm, Galway, Ireland) and remained seated for a 15‐min rest period in an ambient temperature room (∼21°C). At the end of this period, baseline measurements were obtained, including automated brachial blood pressure (in triplicate; M6 Comfort; Omron Healthcare, Kyoto, Japan), tympanic temperature (ThermoScan 7; Braun, Kronberg, Germany), perceptual measures of thermal sensation (1–13 scale, unbearably cold–unbearably hot) and discomfort (1–10 scale, comfortable–extremely uncomfortable; adapted from Gagge et al. (1967)), and capillary blood samples collected in triplicate for the determination of haematocrit (Hct; microcentrifugation: 5 min at ∼12,000 *g*; HCT NXT; Hawksley, Brighton & Hove, UK; read using a micro‐haematocrit reader) and haemoglobin concentration ([Hb]; ABL80 CO‐OX; Radiometer, Copenhagen, Denmark).

Participants then entered the FIR sauna, where *T*_REC_, *T*_SK_ and HR were recorded continuously throughout the 45‐min exposure. Perceptual measures of thermal sensation and discomfort were obtained at 15, 30 and 45 min. No fluids were consumed during the exposure.

At the conclusion of the exposure, participants returned to the ambient laboratory environment (∼21°C) and were seated while tympanic temperature, blood pressure measurements and capillary blood sampling were repeated within 3 min of sauna exit. Participants then returned to a private area where nude body mass was reassessed, after which ad libitum rehydration was permitted (∼11°C tap water).

Participants subsequently completed a 30‐min seated recovery period. At the end of recovery, final blood pressure measurements and capillary blood samples were obtained. Participants were then de‐instrumented, and the protocol was complete.

### 2.6 Data analysis

Derived variables were calculated using standard approaches. Mean *T*_SK_ was calculated from measurements obtained at the chest, upper arm, thigh and calf using a four‐site weighted formula (Ramanathan, 1964):

$$T_{\mathrm{SK}}=0.3\left(\mathrm{chest}\right)+\;0.3\left(\mathrm{upper}\mathrm{arm}\right)+\;0.2\left(\mathrm{thigh}\right)+\;0.2\left(\mathrm{calf}\right)$$

Sweat loss was calculated from the change in nude body mass, and sweat rate was determined relative to exposure duration. Percentage dehydration was calculated as the change in body mass relative to baseline. Mean arterial blood pressure (MAP) was estimated as the sum of two‐thirds diastolic blood pressure (DBP) and one‐third systolic blood pressure (SBP). Plasma volume changes were estimated from Hct and [Hb] using the Dill and Costill equation (1974):

$$\%\Delta \mathrm{PV}=\left(\frac{Hb_{pre}}{Hb_{post}}\right)\times \left(\frac{1-Hct_{post}}{1-Hct_{pre}}\right)\;-1\times 100$$

### 2.7 Statistical analyses

All data were analysed using GraphPad Prism (version 11.0.0, GraphPad Software, Boston, MA, USA). Data were assessed for normality by visual inspection of *Q–Q* plots. One‐way repeated‐measures ANOVA was used to assess the effect of time for normally distributed continuous variables collected at more than two time points. Thermal sensation and discomfort were treated as ordinal variables and analysed using Friedman's test. In cases where data were missing, linear mixed‐effects models were used to account for incomplete observations. Continuously monitored variables (i.e., *T*_REC_, HR) were averaged into 5‐min intervals before analysis. Where a main effect of time was observed, *post hoc* comparisons were performed using Dunnett's test, with baseline as the reference time point. For thermal sensation and discomfort, *post hoc* comparisons against baseline were performed using Dunn's multiple comparisons test. Student's paired *t*‐test was used for pre–post comparisons. Continuous data are presented as means ± SD, with 95% confidence intervals where appropriate. Thermal sensation and discomfort are presented as median (interquartile range). Statistical significance was accepted at *P* < 0.05.

## 3 RESULTS

Mean ambient temperature and relative humidity within the sauna were 63.9 ± 3.1°C and 19.0 ± 4.5%, respectively. FIR emitters remained active throughout the exposure, with ambient temperature increasing from 60.7 ± 2.6°C at baseline to 65.0 ± 2.1°C at the end of heating (∆: +4.2°C [95% CI: 0.5, 7.9]; *P* = 0.025), whereas relative humidity increased from 12.4 ± 2.5% to 23.0 ± 2.6% (∆: +10.7% [95% CI: 9.0, 12.5]; *P* < 0.001), likely reflecting progressive moisture accumulation from evaporated sweat and exhaled air within the enclosed environment. Mean tolerance time was 44.4 min. Two participants terminated the exposure early at 40 and 43 min, respectively, due to extreme thermal discomfort.

### 3.1 Thermoregulatory responses

**FIGURE 1:**
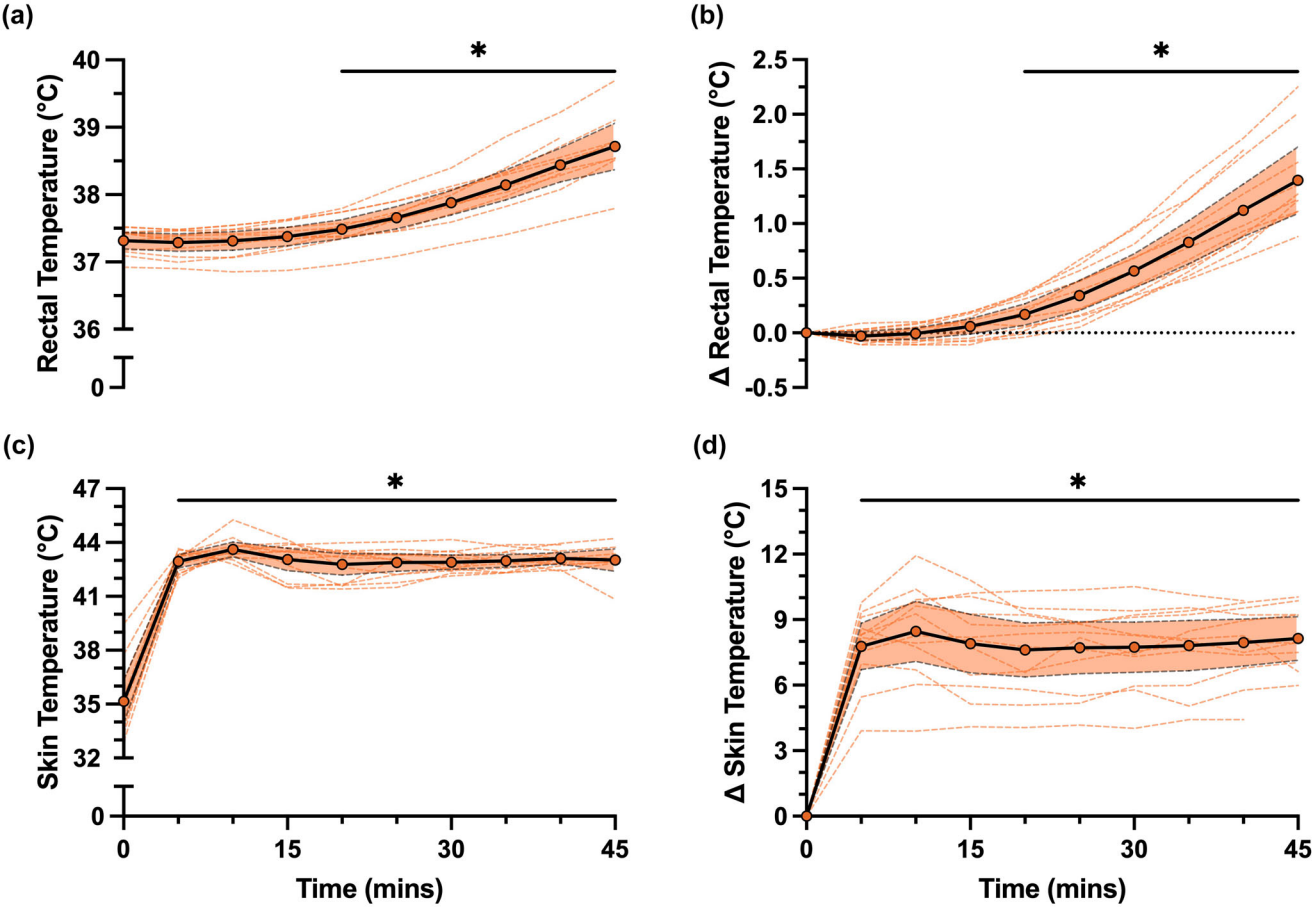
Thermoregulatory responses during FIR sauna exposure. Absolute rectal temperature (a), change in rectal temperature (b), absolute mean skin temperature (c), and change in mean skin temperature (d) across the 45‐min FIR sauna exposure (*n* = 12 from 0–40 min; *n* = 10 at 45 min). Continuous lines represent the group mean, shaded regions denote 95% confidence intervals, and dashed lines represent individual participant responses. *Significant difference from 0 min time point, assessed using repeated‐measures analysis with Dunnett's *post hoc* comparisons.

*T*_REC_ increased over time during FIR sauna exposure (Figure 1a,b), rising from 37.3 ± 0.2°C at baseline to 38.7 ± 0.5°C at the end of heating (∆: +1.4°C [95% CI: 0.9, 1.8]; *P* < 0.001). *T*_REC_ was significantly elevated above baseline from 20 mins (∆: +0.2°C [95% CI: 0.0, 0.3]; *P* = 0.019), and remained elevated throughout the remainder of the heating period. Tympanic temperature also increased following FIR sauna exposure (baseline: 37.1 ± 0.3°C; post‐heating: 39.1 ± 0.5°C; ∆: +2.1°C [95% CI: 1.8, 2.4]; *P* < 0.001); however, absolute values were 0.4 ± 0.5°C higher than those observed for *T*_REC_ at the end of heating.

Mean skin temperature increased rapidly during the initial phase of FIR sauna exposure (Figure 1c,d), rising from 35.2 ± 1.9°C at baseline to 42.9 ± 0.6°C at 5 min (∆: +7.8°C [95% CI: 5.9, 9.7]; *P* < 0.001), and remained elevated thereafter with no further change from 5 min to the end of heating (43.0 ± 0.9°C; ∆: +0.1°C vs. 5 min [95% CI: −0.8, 1.1]; *P* > 0.999). Local skin temperature responses at the chest, arm, thigh and calf are presented in Table 1.

**TABLE 1:** Local skin temperature responses during 45 min of far‐infrared sauna exposure.

Time (min)	*T*_CHEST_ (°C)	*T*_ARM_ (°C)	*T*_THIGH_ (°C)	*T*_CALF_ (°C)
**0**	36.3 ± 1.6 (34.2–39.6)	35.3 ± 2.2 (32.6–40.3)	34.6 ± 1.7 (32.3–38.2)	33.7 ± 2.6 (30.6–39.3)
**5**	43.2 ± 1.0 (41.3–44.7)	44.1 ± 0.8 (42.5–45.6)	41.5 ± 0.9 (39.7–42.8)	42.1 ± 1.9 (38.9–45.8)
**10**	43.5 ± 1.1 (42.1–46.5)	44.6 ± 1.5 (43.5–48.9)	42.8 ± 0.5 (42.2–43.6)	43.1 ± 1.0 (41.2–44.7)
**15**	42.9 ± 1.0 (40.8–43.7)	43.8 ± 1.6 (41.4–47.3)	42.4 ± 1.2 (40.4–43.6)	42.8 ± 1.3 (40.3–44.6)
**20**	42.5 ± 0.9 (41.0–43.8)	43.3 ± 1.1 (41.4–44.9)	42.4 ± 1.2 (40.7–43.8)	42.7 ± 1.2 (40.6–44.2)
**25**	42.9 ± 0.8 (41.5–44.4)	43.3 ± 1.1 (41.5–44.8)	42.5 ± 0.9 (41.5–44.0)	42.7 ± 1.0 (40.9–44.5)
**30**	42.7 ± 0.6 (41.9–43.9)	43.3 ± 0.8 (42.1–44.7)	42.8 ± 0.8 (41.8–44.1)	42.7 ± 1.0 (40.9–44.5)
**35**	42.8 ± 0.5 (41.9–43.4)	43.3 ± 0.7 (42.3–44.6)	42.8 ± 0.7 (42.2–43.8)	43.0 ± 1.0 (41.3–44.6)
**40**	42.9 ± 0.6 (42.1–44.2)	43.5 ± 0.8 (42.3–44.9)	42.8 ± 0.4 (42.3–43.7)	43.2 ± 0.8 (42.0–44.6)
**45**	43.0 ± 0.5 (42.4–44.1)	43.4 ± 1.3 (41.1–45.5)	42.5 ± 0.9 (40.0–43.1)	42.9 ± 1.6 (38.8–44.4)

*Note*: Values are presented as mean ± SD (range), where range represents the lowest and highest individual values recorded at each time point. *n* = 12 from 0–40 min; *n* = 10 at 45 min.

Nude body mass decreased following sauna exposure (∆: −1.0 kg [95% CI: −0.8, −1.3]; *P* < 0.001), corresponding to a sweat rate of 1.4 ± 0.5 L h^−^^1^. Percentage dehydration was 1.5 ± 0.6%, with 2/12 participants exceeding 2% dehydration.

### 3.2 Cardiovascular responses

**FIGURE 2:**
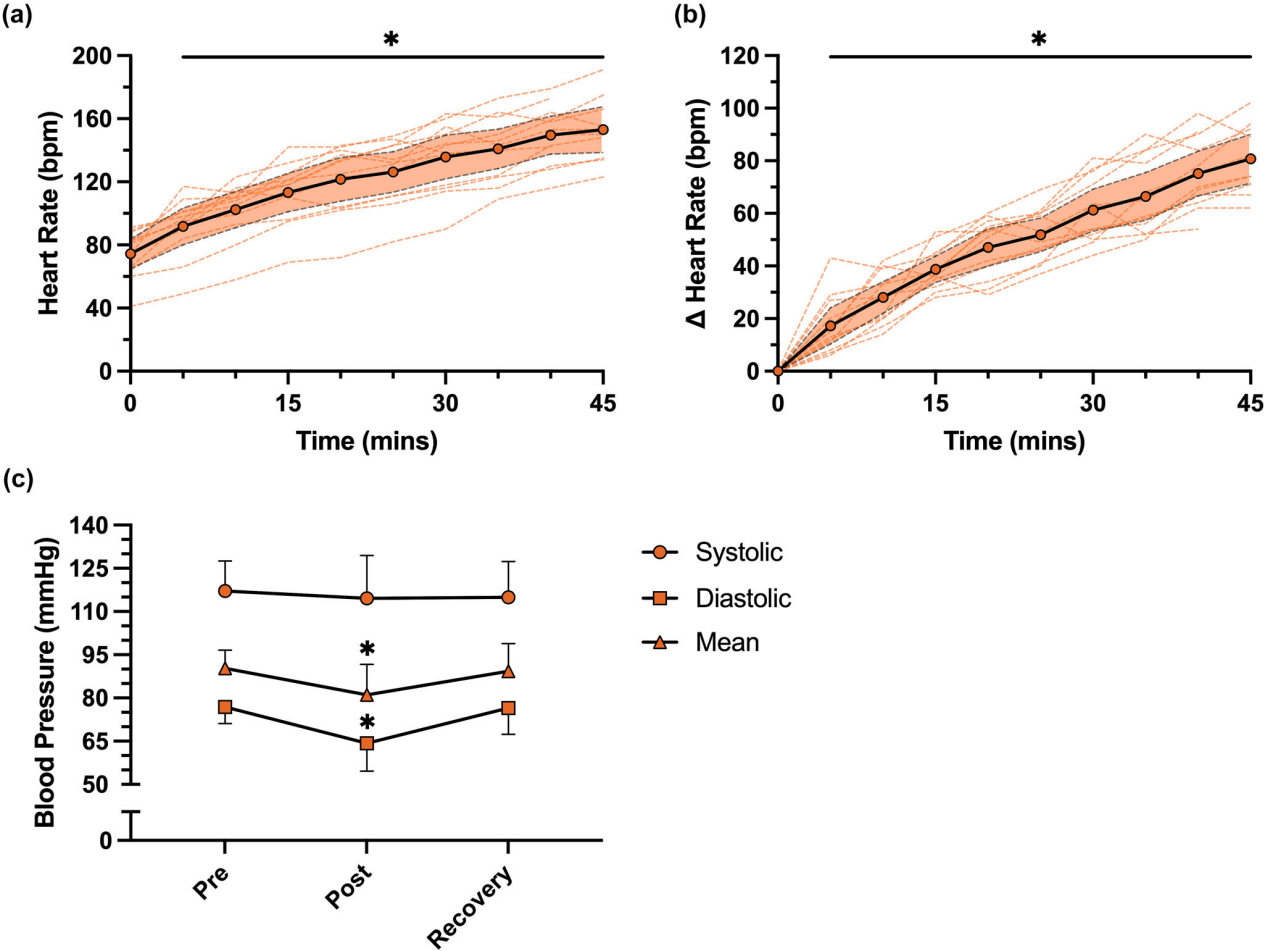
Cardiovascular responses during FIR sauna exposure. (a,b) Absolute heart rate (a) and change in heart rate (b) across the 45‐min FIR sauna exposure (*n* = 12 from 0–40 min; *n* = 10 at 45 min). (c) Systolic, diastolic, and mean arterial pressure responses at pre‐, post‐ and recovery time points (*n* = 12 for all time points). Continuous lines represent the group mean, shaded regions denote 95% confidence intervals, and dashed lines represent individual participant responses (a, b). Data in (c) are presented as means ± SD. *Significant difference from 0 min or ‘Pre’ time point, assessed using repeated‐measures analysis with Dunnett's *post hoc* comparisons against baseline.

HR increased during FIR sauna exposure (Figure 2a,b), rising from 74 ± 15 bpm at baseline to 153 ± 20 bpm at the end of heating (∆: +81 bpm [95% CI: 67, 95]; *P* < 0.001; Figure 2a).

MAP was reduced from 90 ± 6 mmHg at baseline to 81 ± 11 mmHg immediately post sauna (∆: −9 mmHg [95% CI: −2, −16]; *P* = 0.012) before returning to 89 ± 10 mmHg by the end of recovery (∆: −1 mmHg vs. baseline [95% CI: −5, 3]; *P* = 0.755; Figure 2c). This reduction was driven primarily by a decrease in DBP, which fell from 77 ± 6 mmHg at baseline to 64 ± 10 mmHg immediately post sauna (∆: −13 mmHg [95% CI: −8, −18]; *P* < 0.001), with no meaningful change in SBP (*P* = 0.707).

### 3.3 Haematological responses

Hct increased from 42.0 ± 3.5% at baseline to 44.7 ± 3.9% immediately post sauna (∆: +2.6% [95% CI: 1.6, 3.7]; *P* < 0.001) before returning to 42.8 ± 3.8% by the end of recovery (∆: +0.8% vs. baseline [95% CI: −0.3, 1.9]; *P* = 0.185). Similarly, [Hb] increased from 14.5 ± 1.6 g dL^−^^1^ at baseline to 15.6 ± 1.6 g dL^−^^1^ immediately post sauna (∆: +1.2 g dL^−^^1^ [95% CI: 0.6, 1.7]; *P* < 0.001) and returned to baseline values during recovery (14.7 ± 1.6 g dL^−^^1^; ∆: +0.3 g dL^−^^1^ vs. baseline [95% CI: −0.2, 0.8]; *P* = 0.300).

Consequently, plasma volume contracted immediately post sauna (∆: −11.6% [95% CI: −15.9, −7.2]; *P* < 0.001) and returned to near‐baseline levels by the end of recovery (∆: −2.9% [95% CI: −7.7, 1.9]; *P* = 0.256).

### 3.4 Perceptual responses

**FIGURE 3:**
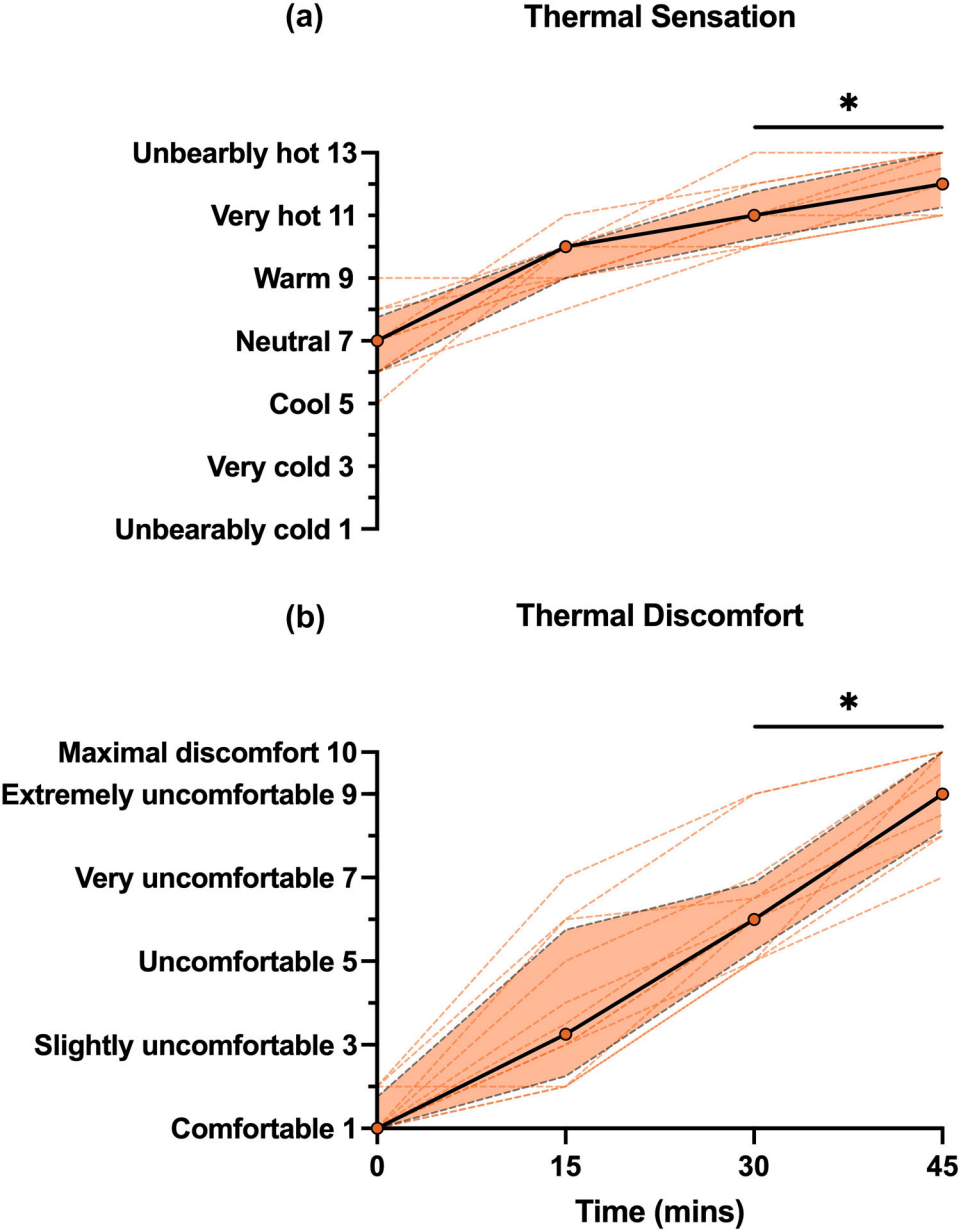
Thermal perceptions during FIR sauna exposure. Thermal sensation (a) and thermal discomfort (b) responses across the 45‐min FIR sauna exposure (*n* = 12 for all time points). Continuous lines represent the group median, shaded regions denote interquartile range, and dashed lines represent individual participant responses. *Significant difference from 0 min time point, assessed using Dunn's *post hoc* comparisons following Friedman's test.

Thermal sensation increased during FIR sauna exposure (Friedman's statistic = 34.8, *P* < 0.001), rising from 7 [IQR: 6, 8] at baseline to 12 [IQR: 11, 13] at the end of heating (‘extremely hot’; Dunn's *post hoc* comparison vs. baseline, *P* < 0.001; Figure 3a). Thermal discomfort also increased (Friedman's statistic = 35.7, *P* < 0.001), rising from 1 [IQR: 1, 2] at baseline to 9 [IQR: 8, 10] at the end of heating (‘extremely uncomfortable’; Dunn's *post hoc* comparison vs baseline, *P* < 0.001; Figure 3b).

## 4 DISCUSSION

The present study demonstrates that FIR sauna exposure at 65°C increases *T*_REC_ in healthy adults, alongside elevations in skin temperature, heart rate and perceptual measures of thermal strain. These findings extend previous work conducted at lower ambient temperatures and indicate that meaningful increases in *T*_REC_ during FIR sauna exposure are achieved at higher thermal loads. The overall physiological response observed here was consistent with that typically reported during established passive heating modalities (including traditional sauna), suggesting that FIR sauna can provide a comparable passive heating stimulus.

### 4.1 Temperature‐dependent increases in rectal temperature during FIR sauna exposure

The present findings demonstrate that increases in *T*_REC_ during FIR sauna exposure are dependent on the thermal load imposed. Whereas previous research has reported no meaningful increase in core temperature (rectal and intestinal) during exposures at 45°C (Reed et al., 2025), or protocols progressing from 45°C to 65°C (Atencio et al., 2025), the present data show that 45 min at 65°C provides a sufficient stimulus to elicit a clear rise in *T*_REC_, with elevations evident after ∼20 min of exposure.

A likely mechanistic explanation relates to the overall balance of heat exchange. FIR delivers energy via radiation, which is primarily absorbed within superficial tissues (i.e., skin and subcutaneous layers) (Vatansever & Hamblin, 2012). At lower ambient temperatures, this localised heat gain may be effectively dissipated through increases in skin blood flow and evaporative cooling, thereby limiting whole‐body heat storage and preventing a meaningful rise in core temperature (Brotherhood, 2008; Gagge & Gonzalez, 2010). However, at the higher ambient temperature employed in the present study (∼65°C), the capacity for heat dissipation is markedly reduced. As ambient temperature exceeds skin temperature, convective heat exchange favours heat gain, while rising humidity, limited air movement and increasing skin wettedness constrain evaporative efficiency (Gagge & Gonzalez, 2010; Jenkins et al., 2022; Sawka et al., 2011). Under these conditions, the thermal environment exceeded compensable limits for heat loss – reflected by estimated wet‐bulb globe temperatures of ∼40–48°C (notwithstanding assumptions in approximating globe and wet‐bulb temperature) (Budd, 2008) – such that net heat gain could not be fully offset by evaporative cooling, thereby favouring progressive heat storage (Brotherhood, 2008; Cheung et al., 2000). In this context, the combined effects of radiative input, convective heat gain and reduced evaporative capacity shift the net heat balance towards progressive whole‐body heat storage. Consequently, heat absorbed at the periphery is less readily dissipated and more effectively transferred to the central circulation, promoting the observed rise in *T*_REC_ (Sawka et al., 2011). Taken together, these findings indicate that the effectiveness of FIR sauna in elevating *T*_REC_ is governed by the magnitude of the thermal load, rather than the modality itself.

### 4.2 Systemic responses to FIR sauna (at 65°C) are comparable to established passive heating modalities

The physiological strain we observed is comparable to that reported during established passive heating modalities. *T*_REC_ increased by ∼1.4°C over the 45‐min exposure, which is within the range typically observed during hot water immersion (∼1.1–1.5°C in ∼40°C water – responses dependent on immersion depth; Campbell et al. 2022; Atencio et al. 2025) and traditional sauna (∼1–2°C; Leppäluoto et al. 1986). Similar elevations in core temperature have been reported in passive heating protocols that successfully induce heat acclimation adaptations, including plasma volume expansion and improved thermoregulatory function (Kissling et al., 2022). The high thermal load observed in the present study was accompanied by marked elevations in skin temperature. Mean skin temperature reached 43°C, which is ∼1–3°C higher than values commonly reported during traditional Finnish sauna exposure protocols (Leppäluoto, 1988; Sohar et al., 1976; Vesala, 1996). Local skin temperatures were also high, with several individual values exceeding 43°C (Table 1). Although we cannot exclude the possibility that direct FIR heating of the thermistor or adhesive interface contributed, at least in part, to these values, the recorded skin temperatures are practically relevant given that thermal discomfort and cutaneous injury risk are time–temperature dependent, with pain often reported >43°C (Martin & Falder, 2017). No participant reported a burning sensation or visible skin injury; however, these findings highlight the importance of skin temperature, perceptual tolerance and participant monitoring when applying high‐temperature FIR sauna protocols, particularly in individuals predisposed to impaired thermoregulation.

These high thermal loads were also accompanied by a marked cardiovascular and haematological responses. HR rose to ∼150 bpm and MAP was reduced post‐exposure, driven by a decrease in DBP. Comparable heart rate responses and reductions in peripheral vascular resistance have been reported during passive heating and heat acclimation protocols, reflecting the cardiovascular adjustments required to support increased skin blood flow and heat dissipation (Crandall & Wilson, 2011; Hannuksela & Ellahham, 2001; Périard et al., 2016). These haemodynamic responses increase vascular shear stress, a key stimulus underpinning the vascular adaptations observed following repeated passive heat exposure or ‘heat therapy’ (Brunt et al., 2016; Green et al., 2010). In parallel, plasma volume contracted by ∼12% alongside sweat losses of ∼1.4 L h^−^^1^, further supporting the presence of a systemic heat stimulus. Acute plasma volume reductions (∼11%; Stephenson & Kolka 1988) and sweat rates (∼1.5 L h^−^^1^; Klous et al. 2021) of a similar magnitude have been reported during passive heating interventions that subsequently elicit plasma volume expansion with repeated exposure (Jenkins Killick et al., 2025; Kissling et al., 2022).

Notably, the thermal stimulus required to elicit these responses was associated with substantial perceptual strain. Thermal sensation and discomfort reached ‘extremely hot’ and ‘extremely uncomfortable’ by 45 min, with two participants unable to complete the protocol. This highlights a trade‐off between achieving a sufficient thermal stimulus and maintaining tolerability. While lower‐temperature FIR protocols (∼45°C) are better tolerated, they appear insufficient to meaningfully elevate core (intestinal) temperature (Reed et al., 2025). Accordingly, shorter exposures or alternative temperature–time combinations may achieve comparable physiological responses with improved tolerability. Taken together, these findings indicate that FIR sauna exposure at 65°C elicits thermoregulatory, cardiovascular, haematological and perceptual responses consistent with those observed in established passive heating modalities.

### 4.3 Perspectives and practical implications

That FIR sauna, when delivered at a sufficiently high thermal load, was able to deliver increases in *T*_REC_, alongside accompanying cardiovascular and haematological responses, indicates that the modality may be capable of providing the thermal stimulus typically required to induce heat acclimation. From a practical perspective, FIR sauna may represent an alternative to exercise‐based heat acclimation protocols, which can be limited by logistical, physical and economic constraints (as reviewed in Heathcote et al., 2018; Jenkins, Edgett et al., 2025). Passive heat acclimation via traditional sauna has been shown to induce a heat‐adapted phenotype, including plasma volume expansion (Stanley et al., 2015), and attenuate the detrimental effects of heat stress on physiological function and exercise performance (Ashworth et al., 2023), suggesting that FIR sauna may also provide a viable means of delivering this stimulus. Furthermore, repeated exposure to elevations in core temperature, coupled with transient plasma volume contraction and fluid regulatory stress, has been linked to longer‐term haematological adaptations, including potential increases in haemoglobin mass (Jenkins, Edgett et al., 2025; Lundby & Robach, 2025). Indeed, we have demonstrated that 5 weeks of passive heat exposure via hot water immersion was sufficient to increase haemoglobin mass and ${\dot{V}}_{O_{2}\max}$ in well‐trained runners (Jenkins Killick et al., 2025); whether FIR sauna can elicit comparable adaptations remains to be determined, but it seems likely.

In addition to these physiological considerations, the operational cost of FIR sauna use may be relatively modest. Based on the rated power output of the unit used here (2.65 kW), a 1 h pre‐heating period followed by 45 min of exposure would require up to ∼4.6 kWh if operated continuously at full power, equating to ≤£1.15 per session at current UK electricity costs (Ofgem, 2026). This corresponds to an estimated carbon cost of ≤1 kg CO_2_ per session based on typical UK grid emission factors (NESO, 2026). These estimates suggest that FIR sauna may offer a relatively low‐cost and scalable means of delivering a meaningful heat stimulus in applied settings. Although FIR sauna has typically been positioned as a wellness or recovery modality, the present findings suggest it may have broader utility as a heat‐based intervention for inducing physiological strain relevant to heat acclimation and heat therapy.

### 4.4 Limitations

A limitation of the present study is that the relative contributions of different heat transfer pathways cannot be isolated. While FIR saunas deliver energy via radiation, the high temperatures achieved here would also promote convective heat gain once ambient temperature exceeded skin temperature. Conductive heat exchange may also have contributed to a lesser extent (e.g., via contact with heated surfaces), although this was likely minimal under the conditions employed. As such, the observed physiological responses reflect the combined effects of these mechanisms, and the independent contribution of radiative heat transfer cannot be determined. A further limitation relates to the measurement of skin temperature during FIR sauna exposure. Surface thermistors were used to record local skin temperature, from which mean skin temperature was calculated. Given the radiant nature of FIR heating, we cannot exclude the possibility that direct heating of the thermistor or adhesive interface may have contributed, at least in part, to the high local skin temperatures reported in Table 1.

Finally, the present findings should be interpreted in the context of the young, healthy participants studied. Although FIR sauna exposure at 65°C provided a sufficient stimulus to increase *T*_REC_, this was accompanied by very high thermal sensation and discomfort. Therefore, the applicability of this protocol to other populations remains uncertain, particularly in individuals predisposed to impaired thermoregulation and in clinical cohorts who may stand to benefit most from heat therapy interventions. Future work should determine whether alternative temperature–duration combinations can achieve comparable increases in core temperature with improved tolerability and greater applicability to these groups.

### 4.5 Conclusions

FIR sauna exposure at 65°C increased *T*_REC_ and elicited thermoregulatory, cardiovascular and perceptual responses consistent with those observed during traditional sauna exposure. These findings demonstrate that FIR sauna can provide an effective passive heating stimulus for increasing core temperature when sufficient thermal load is achieved. Accordingly, FIR may have potential as a heat therapy intervention or as a modality for heat acclimation.

## AUTHOR CONTRIBUTIONS

All authors contributed to the design and writing of the manuscript. All authors approved the final version of the manuscript and agree to be accountable for all aspects of the work in ensuring that questions related to the accuracy or integrity of any part of the work are appropriately investigated and resolved. All persons designated as authors qualify for authorship, and all those who qualify for authorship are listed.

## CONFLICT OF INTEREST

The sauna used in this study was provided to Cardiff Metropolitan University by Clearlight Saunas. The company had no role in the design, conduct, analysis, or interpretation of the study. No financial compensation was received by the authors, and the authors declare no other competing interests or commercial relationships with the company.

## GENERATIVE AI STATEMENT

During the preparation of this manuscript, the authors used ChatGPT (OpenAI) to support language editing, clarity, structure and refinement of author‐generated text. The tool was not used to generate original scientific ideas, analyse data, create figures or produce references. All AI‐assisted suggestions were reviewed, edited and approved by the authors, who take full responsibility for the content of the submitted manuscript.

## Data Availability

The datasets generated and analysed during the current study are available from the corresponding author on reasonable request.
